# Recombinant VAA-I from *Viscum album* Induces Apoptotic Cell Death of Hepatocellular Carcinoma SMMC7721 Cells

**DOI:** 10.3390/molecules171011435

**Published:** 2012-09-26

**Authors:** Xueliang Yang, Shuang Jiang, Yahui Liu, Ping Zhang, Shuli Xie, Guangyi Wang

**Affiliations:** 1 Department of Hepatobiliary and Pancreatic Surgery, the First Clinical Hospital, Jilin University, Jilin 130021, China; 2 Department of General Surgery, the Affiliated Hospital, Beihua University, Jilin 132000, China; 3 The College of Pharmacy, Beihua University, Jilin 132013, China

**Keywords:** *Viscum album*, rVAA-I, apoptosis, phosphoinositide 3-kinase

## Abstract

Researchers have proposed that VAA-I, a specific plant lectin found in *Viscum album*, has therapeutic effects on cancer and autoimmune diseases. VAA-I has shown some promising treatment results in some types of tumor cell lines, especially SMMC-7721 cells (human hepatocellular carcinoma cells). However, few details are known about the mechanism and process of cell death induced by VAA-I in tumor cells. In this study, the cell morphology results showed that SMMC-7721 cells treated with VAA-I exhibited several features typical of apoptotic cell death, which was confirmed by the Caspase inhibition assay. Fluo-3-acetoxymethyl ester (AM) fluorescence imaging techniques showed that rVAA-I significantly elevated the intracellular calcium level ([Ca^2+^]i) in SMMC-7721 cells. These findings suggest that apoptosis may play the most important role in SMMC-7721 cell death induced by rVAA-I. Finally, in the SMMC-7721 cells treated with rVAA-I, a series of genes in the p38 mitogen-activated protein kinase (MAPK) signaling pathway were expressed differentially, and further found that PI 3-kinase pathway is involved in rVAA-I signal transduction in SMMC-7721 cells.

## 1. Introduction

*Viscum album* agglutinin-I (VAA-I) is a specific plant lectin of approx. 63 kDa that is composed of two distinct subunits, named A chain and B chain [1,2]. The A chain confers protein synthesis inhibitory properties to the VAA-I molecule by acting as a ribosome-inactivating agent. This is due to RNA-glycosidase activity which inhibits N-glycosylation of a single adenine within a universally conserved GAGA sequence on the 28S rRNA [3]. The B chain allows the VAA-I molecule to bind to the terminal galactoside residues on the cell surface. At low concentrations, VAA-I is known to induce protein synthesis, while it inhibits this biological response when used at higher concentrations [4]. The lectin already causes cytotoxic effects in the picogram range after eukaryotic cells are incubated for 24 h in the presence of VAA-I, for example, in the case of K562 (human erythroleukemia) cells or EL-4 (mouse thymoma) cells [5,6]. In PBMC cultures VAA-I also starts to have a cytostatic as well as a cytotoxic effect at concentrations above 10 ng/mL if incubated for 24 h [4]. If the incubation time is shorter, this toxic limit is naturally higher. It could also be proved that the growth-inhibiting effect of mistletoe extracts and VAA-I in different cell cultures *in vitro* can be traced back to the induction of programmed cell death [7]. In cultures of U937 promonocytes, VAA-I causes an increased cytosolic Ca^2+^ concentration which, among other factors, is a sign of apoptosis [8]. Results from the Kaveri group suggested that VA extract-induced endothelial apoptosis may explain the tumor regression associated with the therapeutic use of VA preparations and support further investigations to develop novel anti-angiogenic compounds based on mistletoe compounds [9].

Here, the recombinant VAA-I was expressed with a *Pichia pastoris* system, then we investigated rVAA-I’s effect on the transcriptome of hepatocellular carcinoma SMMC7721 cells, and relevant morphological changes and signal transduction mechanisms.

## 2. Results and Discussion

### 2.1. rVAA-I was Expressed in Pichia pastoris

**Figure 1:**
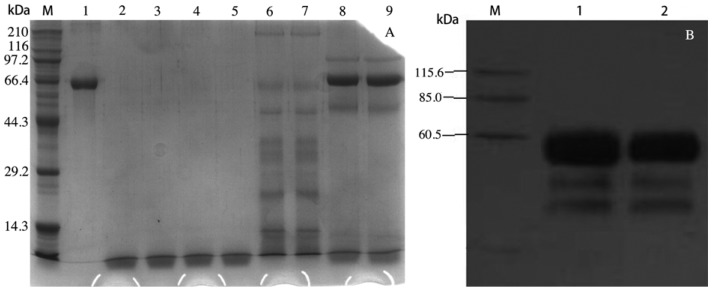
SDS-PAGE analysis of rVAA-I generated from *Pichia pastoris* (**A**). Lane M: Markers lanes, Lane 1: purified VAA-I, Lane 2–5: BMMY culture, Lane 6–7: supernantants of transformed yeast with blank pPICZα-A, Lane 8–9 supernantants of transformed yeast; Western Blotting analysis of rVAA-I generated from *Pichia pastoris* (**B**). Lane M, Markers; Lane 1–2, the induced supernatant of yeast transformants.

The VAA-I gene was amplified by PCR. The PCR product was then introduced into a pPICZα-A™ expression vector through an enzyme site and the recombinant vector was later confirmed by sequencing with 5'AOX1 primers. The sequencing result showed that the nucleic acid sequence was the same as VAA-I. Culture supernatant of the highest expressed transformant was analyzed by SDS-PAGE, which demonstrated an approximate 62 kDa protein consistent with the molecular weight calculated from the amino acid sequence of VAA-I. Such a protein band could not be detected when *Pichia* strain with blank pPICZα-A was induced with methonal. The positive protein band on the SDS–PAGE gel (Figure 1A) was confirmed by Western blotting (Figure 1B).

### 2.2. rVAA-I Induced SMMC7721 Cell Death

**Figure 2:**
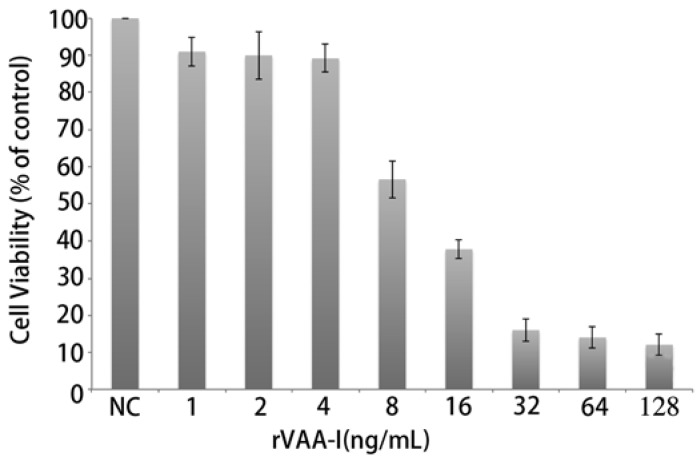
Inhibition of cell growth of SMMC-7721 cells treated with rVAA-I. SMMC-7721 cells treated with a range of rVAA-I concentrations (1.0–128.0 ng/mL) for 24, 36, 48 and 72 h. Viability was measured with MTT reagent after the indicated period of time. Points represent the mean of three similar experiments (n = 3); bars, SE.

Microscopic observations revealed that rVAA-I had a very distinct killing effect on SMMC7721 cells. In addition, the MTT assays showed that addition of rVAA-I (1.0, 2.0, 4.0, 8.0, 16.0, 32.0, 64.0 and 128.0 ng/mL) decreased cell viability of SMMC7721 cells in a dose- and time-dependent manner. SMMC7721 cells were incubated with various concentrations of rVAA-I for 24 h and then the MTT assay was performed to determine the depressive effect of rVAA-I on cell viability (Figure 2). Slight inhibition of viability was detected in cells exposed to 8 ng/mL of rVAA-I, whereas cell viability was markedly inhibited in cells treated with 16.0 ng/mL of rVAA-I. These data showed that rVAA-I inhibited SMMC7721 cell viability in a dose- and time- dependent way, with IC50 at 24 h of 16 ng/mL [10].

### 2.3. Apoptosis Plays a Major Role in SMMC-7721 Cell Death Induced by rVAA-I

**Figure 3:**
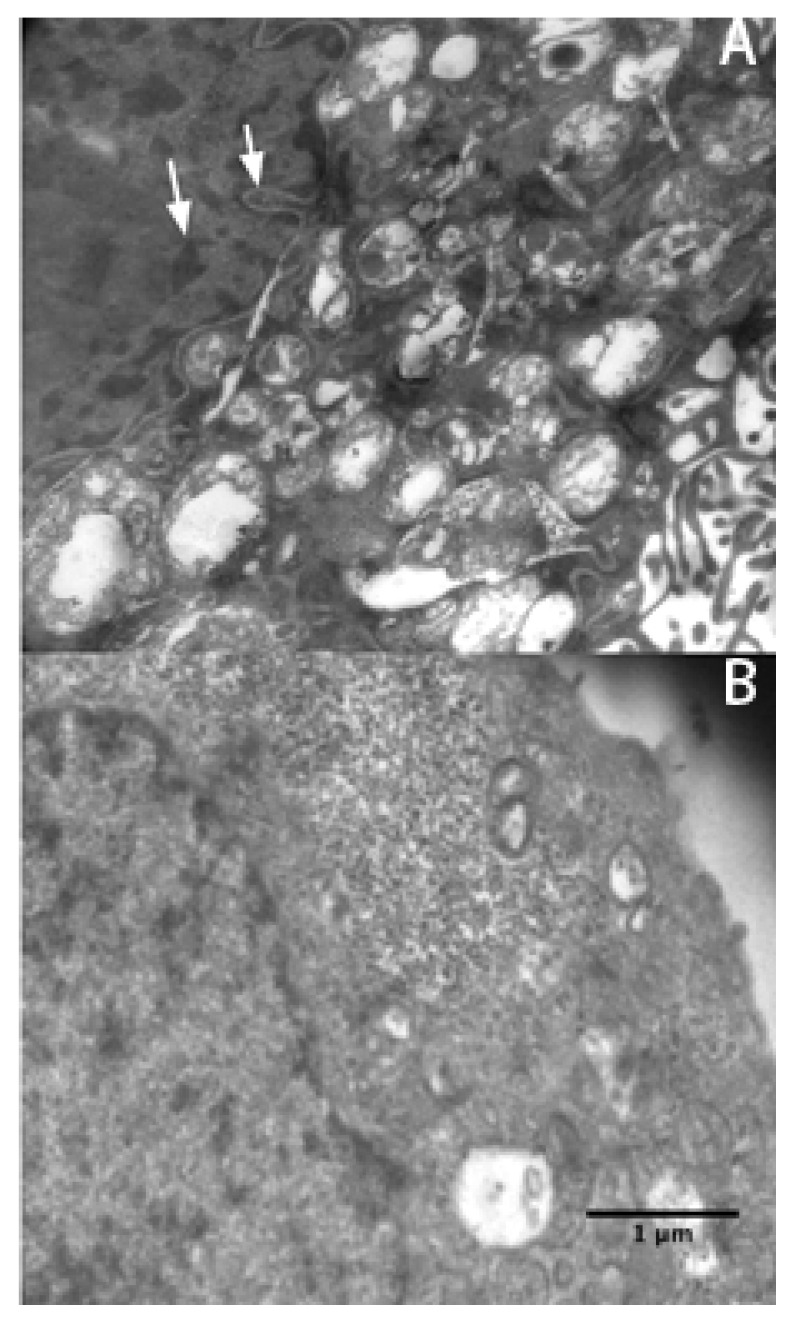
Electron micrographs were taken of SGC7901 cells treated (**A**) and untreated (**B**) with 16 ng/mL rVAA-I. Typical apoptotic morphological changes are denoted by an white arrow. Scale bar: 1 μm.

The extent of rVAA-I-induced cell death was evaluated using flow cytometry (FACSCalibur, BD Biosciences). SMMC7721 cells were treated with different concentrations of rVAA-I for 24 h in medium. Next, apoptosis was measured by Annexin V-FITC (early apoptosis) and propidium iodide (PI, late apoptosis) staining and analysis. Increases in apoptotic cells relative to the control were observed in cells exposed to each concentration of rVAA-I. Incubation with rVAA-I for 24 h induced apoptosis dose dependently in SMMC7721 cells cultures, reaching a 30% total apoptotic (early and late) rate for 8 ng/mL rVAA-I and a 44% total apoptotic rate for 16 ng/mL rVAA-I. SMMC7721 cells exposed to 32 ng/mL rVAA-I resulted in a 51% total apoptotic rate. In comparison, 8% total apoptotic cells were found in the untreated control, which most likely resulted from the isolation procedure (*p* < 0.05). This result suggests that exposure to rVAA-I could induce SMMC7721 cell apoptosis. Micrographs of SMMC7721 cells treated with 16 ng/mL rVAA-I showed karyopyknosis, chromatic agglutination, and nuclear fragmentation (white arrows) (Figure 3A). All of these features are typical apoptotic morphological changes, especially condensation and shrinkage of nuclei. These latter changes were confirmed by Hoechst 33342 staining (unpublished data). 

**Figure 4:**
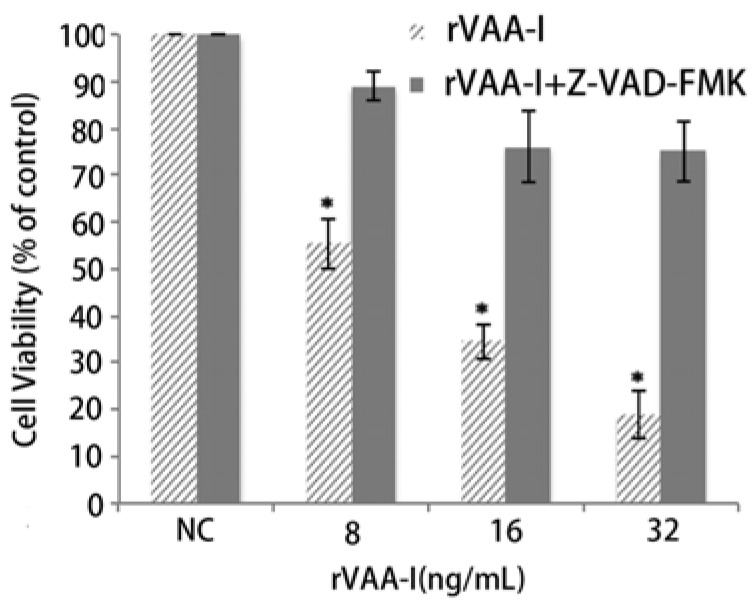
Effect of caspases in SMMC-7721 cells treated with rVAA-I. SMMC-7721 cells untreated or treated with 50 μm Z-VAD-FMK for 30 min, and then treated with 8, 16, 32 ng/mL rVAA-I. The result measured with MTT. Standard error represents four independent experiments.

It is widely accepted that apoptosis is mediated by caspase activity, the specific role of caspases is debatable [11,12,13]. A caspase inhibition assay was performed to examine the involvement of caspase in rVAA-I-induced cell death. The caspase inhibitor z-VAD-fmk (50 mM) did significantly affect the cell viability of rVAA-I-treated SMMC-7721 cells (Figure 4). These results proved that rVAA-I-induced cell death depends on caspase activated apoptosis. 

### 2.4. rVAA-I Triggers Calcium and Cytochrome c Release

**Figure 5:**
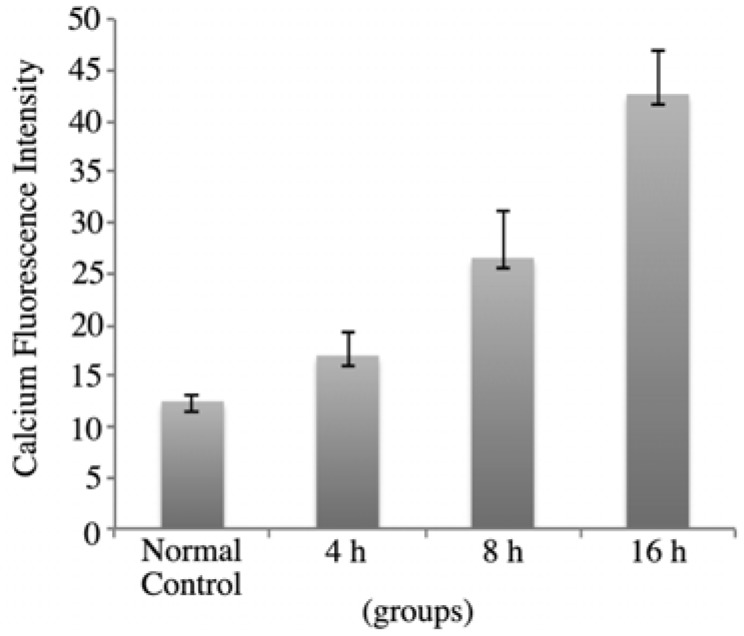
rVAA-I could trigger calcium release in SMMC-7721 cells for 4, 8 and 16 h.

Chromatin condensation is one of the hallmarks of apoptosis, and it is regulated by specific genes. Taking all of our results into consideration, we propose that rVAA-I induces apoptosis. In addition, [Ca^2+^]i increased after treatment with rVAA-I in this study. Ca^2+^ is a ubiquitous second messenger that mediates a wide range of cellular responses, such as contraction, fluid and electrolyte secretion, exocytosis, gene transcription, and apoptosis. The ability, simultaneously controlling multiple processes, occurs when carefully modulating Ca^2+^ signals. This is also reported not only over time but in different subcellular regions. Recently, it was suggested that nuclear and cytosolic calcium are regulated independently. Several lines of evidence demonstrate that increases in Ca^2+^ within the nucleus have specific biological effects that differ from the effects of increases in cytosolic Ca^2+^. The Ca^2+^ indicator Fluo-3/AM assay provided further proof that rVAA-I could induce SMMC7721 cells apoptosis. As is shown in Figure 5, in SMMC7721 cells treated with 16 ng/mL rVAA-I for 4 h, the calcium fluorescence intensity was 17.36 ± 2.22. After treatment for 8 and 16 h, the fluorescence intensity was 26.99 ± 3.69 and 42.48 ± 4.32, respectively. These values were significantly from those of the controls (*p *< 0.05). These results indicated that rVAA-I could trigger calcium release and thereby cause intracellular calcium overload. 

**Figure 6:**
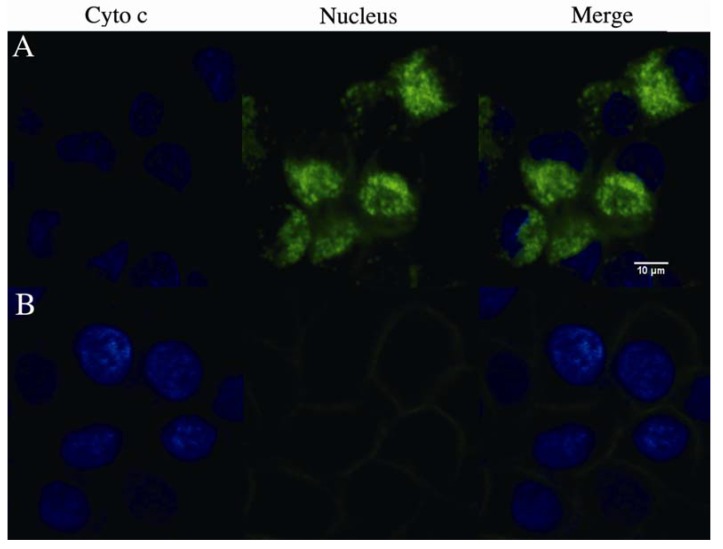
Effect of rVAA-I on the cytochrome c release from mitochondria. (**A**) SMMC-7721 cells with confocal medium and (**B**) cells treated with of 16 ng/mL rVAA-I for 24 h were fixed and labeled for cytochrome c (green) and nucleus (blue). Images were obtained using confocal laser scanning microscopy.

Next we analyzed the cytochrome c release from mitochondria using confocal microscopy. As shown in Figure 6, cytochrome c (green) release was observed significantly in SMMC-7721 cells treated with 16 ng/mL rVAA-I for 18 h. However, cytochrome c release in non-treated control cells was not observed. Therefore, we conclude that cytochrome c is released from mitochondria to cytosol in rVAA-I-treated SMMC-7721 cells.

### 2.5. PI 3K Pathway May Play an Important Role in the Process of Cell Death

Genome-wide transcriptional profiling of SMMC7721 cell has demonstrated that extensive gene expression occurs during the cell was treated with rVAA-I. To investigate the possible gene expression change in our rVAA-I induced apoptosis cell model, we performed a gene chip study by using the Affymetrix probe dataset which includes a total of 22,000 probes. We found when compared with probes in the control group, 85 probes increased and 42 probes decreased in the rVAA-I treated group. 

T-test correction of the randomized variance model was performed to determine genes that were expressed separately and differentially. As a result, total 130 genes that had a p-value and the false discovery rate (FDR) less than 0.05 were declared to be significantly expressed. The gene expression value per group was the geometric mean of the Robust Multichip Average (RMA) normalized gene signals of three samples per group.

The complexity of gene relationship increased with k-core value rank. We wanted to find the main pathways assigned by the maximum numbers of genes in separately k-core and then define the key gene functions at each complexity level of network. For this analysis result, we determined the core functions at the core status of network which have a top k-core level (Figure 2A). We can find that there are many DifGenes in the pathways, such as biosynthesis of steroids, cell adhesion molecules (CAMs), MAPK signaling pathway, terpenoid biosynthesis and synthesis and degradation of ketone bodies.

The gene expression profile in untreated and rVAA-I-treated cells also indicated that rVAA-I had a great impact on some important genes in the MAPK pathway. Some GO Terms associated with the nucleus differed between treated and control cells, including the MAPK signaling pathway (RAP1B, MAPK14, FLNA), apoptosis (SMNDC1), proteasome (PSMA2, PSMA4), riboflavin metabolism (RFK, MTMR6), tryptophan metabolism (FANCL, BRAP), the TGF-β signaling pathway (AMH), the phosphatidylinositol signaling system (IMPA1), and oxidative phosphorylation (PPA2) [14]. The MAPK signaling pathway is involved in a wide range of cellular processes, such as viability, differentiation, transcription regulation, and development. The role of MAPK in apoptosis requires further study. In our study, FLNA, MAPK14, and RAP1B gene expression changed when cells were exposed to rVAA-I. FLNA is a 280-kDa dimer composed of the amino-terminal actin-binding domain, which interacts with a number of proteins with roles in signaling and cytoskeletal reorganization and is regulated by phosphorylation [14,15]. FLNA has been implicated in MAPK signaling induced by a variety of extracellular stimuli, and it can interact with the MAPK kinases MEK1 and MKK4 [16]. It also can be phosphorylated by ribosomal S6 kinase [17], which is an ERK target. RAP1 is a small GTPase that becomes activated downstream from multiple surface receptors via guanine nucleotide exchange factors. It also regulates several basic cellular functions such as adhesion, migration, polarity, differentiation, and growth. Moreover, it has been reported that RAP1 activates the MAP kinase pathway in several cell types [18].

Our data suggest that the MAPK signaling pathway plays a critical role in SMMC7721 cells treated with rVAA-I. We used the KEGG, BioCarta, and GenMAPP databases to identify the gene network and the genes how it regulated. We then used real-time qPCR to validate the microarray results. Three differentially expressed genes (MAPK14, IMPA1, and SMNDC1) in the MAPK signaling pathway was selected for verification by real-time qPCR. In general, these results were consistent with those obtained by the microarray. Similar changes in the expression levels of representative mRNAs were observed using the chip arrays and real-time qPCR. Although the mRNA expression levels were not identical for real-time qPCR and the cDNA microarray, the increases or decreases were parallel between methods. 

Under the experimental conditions used in this study, we observed expression changes in a series of genes that have multiple functions. The microarray study indicated that the biological processes of genes differentially expressed due to rVAA-I treatment included signal transduction and transcription regulation. When we examined the pathways of rVAA-I-induced genes using the MAS 2.0 system, we found that they were mainly involved in MAPK signaling and proteasome and riboflavin metabolism.

The protein encoded by MAPK14 is a member of the MAP kinase family, which acts as an integration point for multiple biochemical signals and is involved in a wide variety of cellular processes as well, such as viability, differentiation, transcription regulation, and development. Using chemical inhibitors in their experiments, Gillis *et al*. determined that the ICP27-mediated activation of p38 signaling is responsible for the observed induction of apoptosis in the induced cell lines [19]. According to Gillis *et al*.’s previous research, arsenite-induced germline apoptosis was blocked in loss-of-function alleles of extracellular signal-regulated kinase (ERK) and p38MAPK cascades. The MAPK signaling pathways are essential for germline apoptosis. In regards to our study, we found that MAPK14 was up-regulated by rVAA-I treatment (unpublished data). The data showed that the p38 MAPK signaling pathway plays an important role in SMMC7721 cell apoptosis. 

### 2.6. Inhibition of Apoptosis by the MAPKs and PI 3-Kinase Inhibitor in the rVAA-I Treated SMMC-7721 Cells

**Figure 7:**
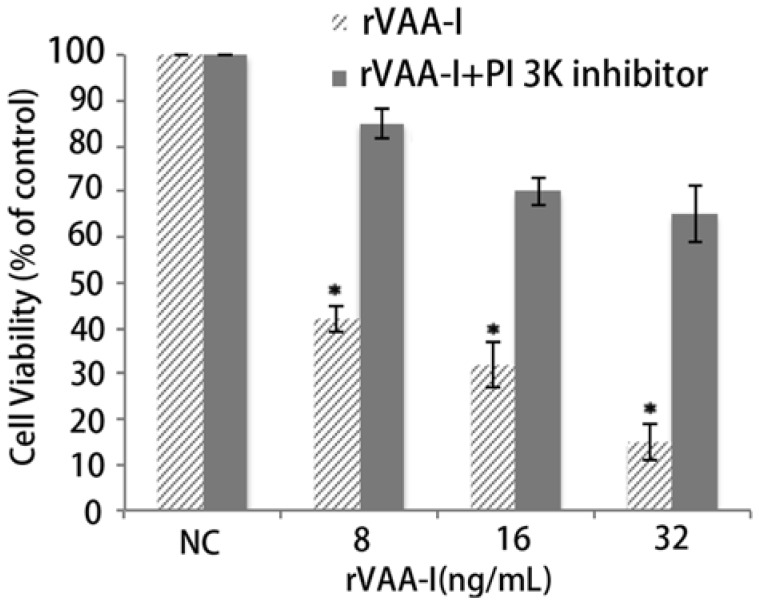
Effect of PI 3K cell signal pathway in SMMC-7721 cells treated with rVAA-I. SMMC-7721cells untreated or treated with 50 μM PI 3K inhibitor (LY294002), and then treated with 8 to 32 ng/mL rVAA-I for 48 h. The result measured with MTT. Standard error represents four independent experiments.

To determine the potential role of MAPKs or PI 3-kinase in the regulation of the apoptosis induced by rVAA-I, we analyzed effects of the p38 inhibitor (SB203580) and competitive PI 3-kinase inhibitor (LY294002) on the apoptosis. After incubating SMMC-7721 cells for 24 h with the mentioned inhibitors, cell were treated with rVAA-I, and the MTT assay results indicated that cell treatment with 5 μM LY294002 significantly abolished the apoptosis induced by rVAA-I (Figure 7). Moreover, a dose-dependent inhibition observed in SMMC-7721 cells. Recently we found that rVAA-I stimulated phospho-ERK (data not shown). This further defined our findings that PI 3-kinase pathway is involved in rVAA-I signal transduction in SMMC-7721 cells.

## 3. Experimental

### 3.1. Recombinant Plasmid Construction and Pichia pastoris Transformation

The rVAA-I gene was synthesized by Sangon Biotech (Shanghai, China) Co., Ltd, KpnI and EcoRI restriction sites were designed for flanking the PCR product at the 5'- and 3'-termini, respectively. To build the expression plasmid, the PCR fragment was cloned into the KpnI/EcoRI site of pPICZα-A™ expression vector (Invitrogen, Camarillo, CA, USA) and transformed into *Pichia pastoris* Gs115 (Mut^+^; Invitrogen). SDS-PAGE gel and the purity of the reFIP after ultrafiltration and ion-exchange chromatograph was about 90% examined by the BCA method.

### 3.2. Media and Culture Conditions for rVAA-I Expression

The *P. pastoris* transformant was cultured in a 100 mL flask containing 1,000 mL BMGY, supplemented with 1% (v/v) glycerol as a carbon source and 200 μg/mL G418 (geneticin) as a selection pressure. Cells were grown at 28 °C and vortexed at 300 rpm until an OD_600_ value reached 15 achieving a 10-fold dilution. The cells were then harvested by centrifugation at 3,000 *g* at 4 °C for 5 min. BMGY was replaced by BMMY containing 0.5% (v/v) methanol, here methanol served to induce AOXI promotor. BMGY and BMMY media were also prepared according to the manufacturer’s instructions (Invitrogen). After 48 h of induction, the supernatant was collected by centrifugation at 4 °C and 12,000 *g* for 20 min. RVAA-I protein was purified by a nickel affinity column Ni-sepharose (GE Lifescience, Piscataway, NJ, USA) and eluted by a gradient of 30–100 mM imidazole. 

### 3.3. Cell Culture and Treatment

The SMMC7721 cell line was obtained from our laboratory. The cells were cultured in IMDM (Hyclone, Logan, UT, USA) containing 10% fetal bovine serum (Sigma, St. Louis, MO, USA), penicillin (100 U/mL), and streptomycin (100 μg/mL). The cells were maintained in a 37 °C, 5% CO2, fully humidified incubator. SMMC7721 cells were incubated for various periods with rVAA-I at different concentrations and then subjected to analysis.

### 3.4. Cell Viability Analysis

Cells were treated with different concentrations of rVAA-I (1.0, 2.0, 4.0, 8.0, 16.0, 32.0, 64.0 and 128.0 ng/mL) for 24 h. At certain times after treatment initiation, cell viability was estimated using a MTT-assay described previously [20]. Briefly, 20 µL of MTT solution (Sigma, 5.0 mg/mL in ddH_2_O) were added to each well. Plates were then incubated for 4 h at 37 °C. Next, 150 µL of isopropanol with 0.04 M HCI were added to each well, and the absorbance of samples was measured at 490 nm. The inhibition ratio was calculated using the equation below, where Ac and At represent the absorbance in the control and treated cultures, respectively:

Inhibition ratio = (Ac − At)/Ac × 100%

### 3.5. Annexin V-FITC/Propidium iodine (PI) FACS

The apoptosis rate of SMMC7721 cells exposed to rVAA-I (0.2, 1.0, and 5.0 μg/mL) for 24 h was determined by flow cytometry using a commercially available Annexin V-FITC apoptosis detection kit (Bipec, Cambridbe, MA, USA). After rVAA-I treatment, cells were collected and washed twice in cold PBS and resuspended in 400 µL binding buffer at a concentration of 1 × 10^6^ cells/mL. The cells were incubated with 5 µL Annexin V-FITC for 15 min and 10 µL PI for the next 5 min at 4 °C in the dark. Finally, samples were analyzed by flow cytometry (BD Biosciences, San Jose, CA, USA) and evaluated based on the percentage of early apoptotic cells (*i.e.*, those that were Annexin V positive and PI negative).

### 3.6. Morphology Assay

Apoptosis was evaluated by observing cell morphology. After treatment with 16 ng/mL rVAA-I for 24 h, the cells were washed and fixed with 2.5% glutaraldehyde in 0.1 M phosphate buffer (pH 7.4), then refixed in 2% osmium tetroxide (Sigma) for 2 h at 4 °C, dehydrated in ethanol at room temperature, and embedded in Quetol 812 (Nisshin EM Co., Tokyo, Japan). Next, 80 nm sections of cells were contrasted with 4% uranylacetate for 15 min and subsequently with lead citrate for 5 min at room temperature. Samples were examined with a Hitachi H-300 transmission electron microscope (Hitachi Ltd., Tokyo, Japan). 

### 3.7. Cytochrome C Release Assay

Assay kits for cytochrome C release apoptosis (Thermo, Franklin, MA, USA) was used to assess the release of cytochrome C from mitochondria to cytosol. The cytochrome c release from mitochondria was analyzed by confocal microscopy. The cytochrome c was labeled with green and nucleus was stained with DAPI (Vector Laboratories, Burlingame, CA, USA). Images were obtained using confocal laser scanning microscopy. 

### 3.8. Determination of Intracellular Calcium Ion Concentration ([Ca^2+^]i)

We used the Ca^2+^ indicator Fluo-3/AM (Biotium, Hayward, CA, USA) to measure [Ca^2+^]i in SMMC7721 cells on chamber slides treated with rVAA-I (16 ng/mL) for 4, 8, and 16 h and then washed with cold PBS. Cells (1 × 10^4^ cells per sample) were loaded with 4 µM Fluo-3/AM for 50 min at 37 °C in the dark and then washed again. Finally, the cells were detected using an FV1000 inverted confocal laser scanning microscope (Olympus). Changes in calcium were recorded as fluctuations in the emitted fluorescence of Fluo-3-complexed calcium at 530 nm (excitation was 488 nm). Fluo-3 fluorescence was analyzed using Image J software (NIH). 

### 3.9. Total RNA Preparation and Microarray Analysis

Cells grown in a monolayer were lysed directly in a culture dish. The media was poured off and TRI reagent (MRC, Cincinnati, OH, USA) was added. Next, the cell lysate was passed several times through a pipette, then total RNA was prepared with TRI reagent according to the manufacturer’s instructions. RNA was treated with DNase, purified by ethanol precipitation, resuspended in DEPC-treated water, and stored at 80 °C. Total RNA was quantified with a spectrophotometer. The total RNA quality was estimated by formaldehyde gel electrophoresis. 

Microarray analysis (22K Human Genome Array) was conducted by Capital Bio Corporation (Beijing, China). The human genome oligonucleotide microarray was prepared by Capital Bio Corporation. The Human Genome Oligo Set Version 2.1 consisting of about 22,000 human genes was purchased from Qiagen Operon Company [21]. Arrays were scanned with a confocal Lux Scanner (Capital Bio Co.), and images were analyzed using Spot Data software (Capital Bio Co.). Probe sets were functionally annotated and grouped according to their biological function using gene ontology (http://www.geneontology.org). The functional analysis to identify the most relevant biological mechanisms, pathways, and functional categories in the data sets of genes selected by statistical analysis was generated through MAS 2.0 (Capital Bio Co.). It combined KEGG, BioCarta, and GenMAPP databases to identify the gene networks and how they are regulated.

### 3.10. Real-Time Quantitative Polymerase Chain Reaction

We confirmed the expression level of genes that were differentially expressed when cells were exposed to rVAA-I using a real-time qPCR. Reserve transcription with 2 µg of total RNA, 1 µg of Oligo (dT) primers, 5 µL 5 × RT buffer, 5 µL 10 mM dNTP mix, 40 U Rnase inhibitor, and 200 U BcaBEST polymerase was performed first to obtain cDNA. Gene speciﬁc primers (Table 1) were used for ampliﬁcation by real-time qPCR on a BIO-RAD Chromo4 Real-Time PCR System using a SYBR Premix Ex Taq^TM^ (Takara Bio Inc., Otsu, Japan) according to the manufacturer’s protocols. Each mRNA expression level was normalized with respect to mRNA expression of glyceraldehyde-3-phosphate dehydrogenase (GAPDH). The data were analyzed using the 2^-Δ^^ΔCt^ method [22], where ∆∆Ct = (CT_Target_ − CT_GAPDH_)_Treatment_ − (CT_Target_ − CT_GAPDH_)_Control_.

### 3.11. Statistics

All results are presented as mean ± SD from triplicate experiments performed in a parallel manner unless otherwise indicated. Statistical analysis was performed by T-TEST and ANOVA ONE WAY. All comparisons were made relative to untreated controls, and *p* < 0.05 was considered significant. The software SPSS 13.0 was used for statistical analysis.

## 4. Conclusions

In the study, we has finished recombinant expression of plant lectin (VAA-I) found in *Viscum album*, and proved that rVAA-I induces an apoptotic cell death in hepatocellular carcinoma SMMC7721 cell via PI 3-kinase pathway.
